# Enduring Neurobiological Consequences of Early-Life Stress: Insights from Rodent Behavioral Paradigms

**DOI:** 10.3390/biomedicines12091978

**Published:** 2024-09-02

**Authors:** Luisa Speranza, Kardelen Dalim Filiz, Pellegrino Lippiello, Maria Grazia Ferraro, Silvia Pascarella, Maria Concetta Miniaci, Floriana Volpicelli

**Affiliations:** 1 Department of Pharmacy, School of Medicine and Surgery, University of Naples Federico II, 80131 Naples, Italy; luisa.speranza@unina.it (L.S.); kardelendalim.filiz@unina.it (K.D.F.); pellegrino.lippiello@unina.it (P.L.); silvia.pascarella@unina.it (S.P.); 2 Department of Molecular Medicine and Medical Biotechnology, School of Medicine and Surgery, University of Naples Federico II, 80131 Naples, Italy; mariagrazia.ferraro@unina.it

**Keywords:** anxiety, cancer, depression, dopamine, glucocorticoids, neurotrophins, neurodegenerative diseases, neuronal plasticity

## Abstract

Stress profoundly affects physical and mental health, particularly when experienced early in life. Early-life stress (ELS) encompasses adverse childhood experiences such as abuse, neglect, violence, or chronic poverty. These stressors can induce long-lasting changes in brain structure and function, impacting areas involved in emotion regulation, cognition, and stress response. Consequently, individuals exposed to high levels of ELS are at an increased risk for mental health disorders like depression, anxiety, and post-traumatic stress disorders, as well as physical health issues, including metabolic disorders, cardiovascular disease, and cancer. This review explores the biological and psychological consequences of early-life adversity paradigms in rodents, such as maternal separation or deprivation and limited bedding or nesting. The study of these experimental models have revealed that the organism’s response to ELS is complex, involving genetic and epigenetic mechanisms, and is associated with the dysregulation of physiological systems like the nervous, neuroendocrine, and immune systems, in a sex-dependent fashion. Understanding the impact of ELS is crucial for developing effective interventions and preventive strategies in humans exposed to stressful or traumatic experiences in childhood.

## 1. Introduction

Stress is the body’s natural response to challenging situations, such as social pressure, threats, or life changes (stressors). It involves various psychological and behavioral reactions to external stressors, disrupting the balance of physiological processes known as homeostasis. The organism’s effort to restore this balance requires the activation of a complex range of responses involving the endocrine, nervous, and immune systems, which constitute the stress response [1]. The brain is a primary mediator and target of stress, playing a pivotal role in stress reactivity, coping mechanisms, and the recovery process [2]. This orchestration occurs through neural networks crucial for emotional processing and cognition, involving the hippocampus, the amygdala, the prefrontal cortex (PFC), and the cerebellum (for details, see [3,4]). These regions can be significantly affected by stressful events, particularly during development [2]. When stress occurs, the brain triggers a complex chain reaction, releasing a cascade of hormones and neurotransmitters, such as glucocorticoids (GCs), norepinephrine, and dopamine (DA), that produce physiological changes aimed at assisting individuals in reacting to or coping with stressors. Sensitivity to stress varies among individuals and can be influenced by several factors, such as perception of stress, past experiences, and ability to manage it. Genetic predispositions, gender differences, and environmental factors also play a role [5,6].

**Figure 1:**
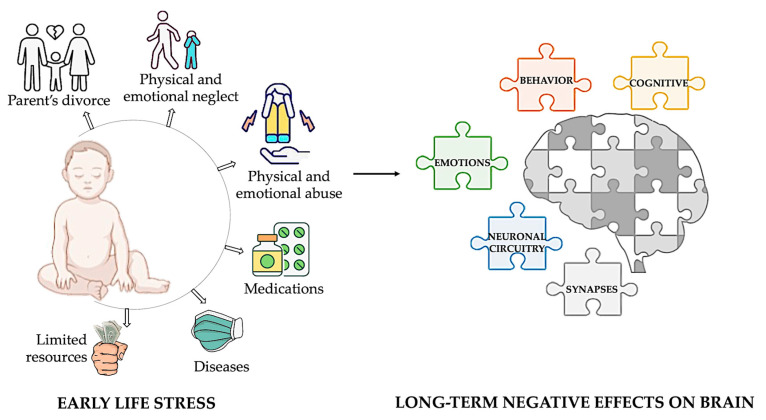
Impact of early-life adversity on brain development and lifelong outcomes. Early-life adversities, including child abuse and neglect, exposure to violence, parental divorce, childhood physical illness, and family economic hardship, profoundly influence brain development. These adversities are strongly associated with alterations in neuronal circuitry and synaptic function, leading to a heightened risk of developing a broad spectrum of behavioral, cognitive, and emotional disorders throughout life. Illustrations in the panel were created with Biorender.com.

Childhood stress, also known as early-life stress (ELS), refers to stress experienced before adulthood. This term encompasses various adverse experiences that a child might face, such as exposure to toxins, nutritional restriction, abuse, neglect, and limited family resources. Prolonged exposure to these situations can have long-term adverse effects on cognitive, emotional, and behavioral processes due to the high rates of synaptic regrowth and remodeling in the brain during infancy and early childhood [7,8,9] (Figure 1).

Exposure to significant stressors increases the risk of developing mental health disorders such as anxiety, depression, and PTSD [10]. Animal studies corroborate these findings, showing similar behavioral effects of adversity, including increased fear, anxiety, depressive-like behavior, and higher drug use, particularly alcohol [11].

Socioeconomic status (SES) significantly impacts various aspects of life, exacerbating stress through factors like financial instability and inadequate healthcare and education. These stressors amplify the effects of ELS on biological and psychological development [12]. Recent research indicates that ELS and SES elevate neuroinflammation, disrupt the HPA axis, and alter immune responses [13,14]. In low SES settings, ELS can affect the hippocampus and emotional regulation, increasing the risk of mood disorders and externalizing behaviors [15,16,17,18].

Evidence from rodents suggests that early-life stressors, such as maternal stress or social isolation, lead to overactivation of the HPA axis and the sympathetic nervous system, establishing pro-inflammatory mechanisms and lasting epigenomic changes that affect brain development and stress responses [1,19,20,21,22]. Epigenetic modifications involve interactions between the environment and the genome, altering phenotypes by either modifying chromatin structure or controlling mRNA translation without changing DNA sequences. Key mechanisms include DNA methylation (addition of methyl groups, typically at CpG sites), histone modifications (methylation, acetylation), and noncoding RNA activity, which influence gene expression by regulating chromatin structure and transcription factor binding [23]. These epigenetic changes mediate effects on neural networks, increasing susceptibility to physical and mental illnesses during adolescence and adulthood [24]. Adverse childhood experiences have also been associated with negative outcomes, including an increased risk of cancer [25].

This review integrates PubMed, Web of Science, and Scopus findings, emphasizing the intricate pathways through which ELS affects brain neurobiology in rodents and humans. By elucidating these mechanisms, the review underscores how ELS heightens vulnerability to cognitive deficits, psychiatric disorders, neurodegenerative diseases, and brain tumors later in life. Understanding and addressing these interactions is crucial for developing effective strategies to support individuals affected by ELS and mitigate its long-term impacts.

## 2. Animal Models of Early-Life Stress

ELS animal models are essential tools in biomedical research. They provide a controlled and ethical means to investigate the complex effects of ELS on brain development and physical and mental health, thus contributing to understanding the biological underpinnings of stress-related disorders. While these models still have limitations, their contributions to science and medicine are invaluable, helping to bridge the gap between basic research and clinical application.

**Figure 2:**
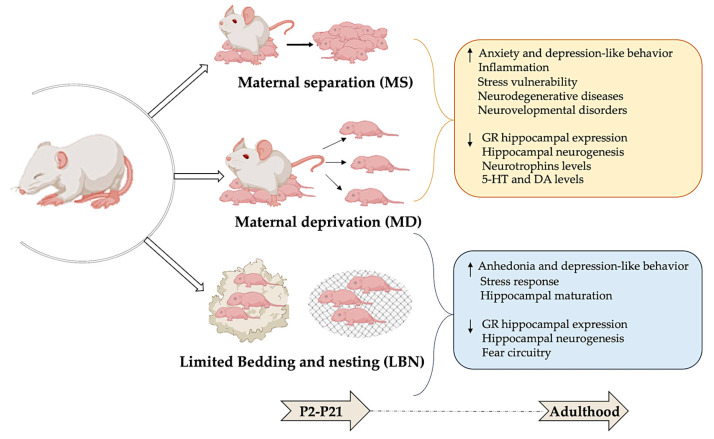
Modeling early-life stress: long-term behavioral and neurobiological effects. Maternal separation (MS), maternal deprivation (MD), and limited bedding and nesting (LBN) are used as rodent models of early-life stress. This figure shows that stress experienced during the postnatal period (P2–21) can lead to significant and lasting changes in both behavioral and neurobiological outcomes in adulthood. Illustrations in the panel were created with Biorender.com.

Three animal models of ELS stemming from the absence of maternal care are currently being utilized in research (Figure 2).

### 2.1. Maternal Separation

One widely used rodent model of ELS is maternal separation (MS), where pups are separated for several hours daily during the first postnatal weeks from the dam but not from the other littermates. Separating rat pups from their mothers for several hours daily during the first postnatal weeks leads to increased anxiety and depressive-like behaviors in adulthood [26]. In adulthood, rats and mice exposed to MS displayed signs of increased non-social fear and anxiety behaviors, including reduced exploration of new environments, less time spent on the open arms of the elevated plus-maze, heightened freezing in open fields, and amplified acoustic startle responses [27].

### 2.2. Maternal Deprivation

Another valuable model for studying ELS is maternal deprivation (MD), where pups are separated from their mother and littermates. Beyond serving as a model of stress, this approach also simulates social isolation, providing a multifaceted perspective on ELS [28]. In their compelling study, Zimmerberg and Sageser (2011) [28] compared the effects of MS and MD on the social behavior of juvenile rats. Their findings revealed that MS induces high stress levels in dams, increasing maternal care upon reuniting with their pups. Conversely, MD resulted in elevated stress levels in the pups, leading to distinct behavioral outcomes [29]. Both MS and MD involve separating the pups from their mother and/or from each other for one or more hours each day during the initial 2 or 3 weeks of life [30,31]. Procedures for separation exhibit wide variations in terms of duration, frequency, and age of pups at separation, leading to diverse data presented in the literature [32,33,34].

Additionally, it is crucial to consider the genetic background and sex of experimental animals. For instance, the C57Bl/6 mouse strain tends to be highly stress-resistant, whereas the BALB/c strain is more stress-sensitive. Furthermore, stress affects males and females differently, rendering specific behavioral tests less suitable for one sex over the other [35].

Moreover, the timing and duration of stressful events in early life are critical factors influencing outcomes [36]. Understanding the complexities of ELS in rodents is crucial, particularly in translational studies, as the timing of stressful postnatal events in rodents may not align with the timing of child abuse, which often persists for several years during childhood [37]. Consequently, questions arise about the direct translational validity of specific ELS models in rodents [38].

Maternal deprivation also serves as a powerful model for studying social isolation. Vaneema et al. have described the effects of post-wearing social isolation and deprivation in non-human primates and rodents [27]. It has been demonstrated that physical contact with cage mates, referred to as “social play”, is generally observed in post-weaned laboratory rats. The “social play” frequency displays an inverted U-shaped curve across development, peaking around 30 days old before declining after sexual maturation [39]. Post-weaning social isolation, in which rats or pups are housed alone, leads to devastating effects compared to isolation in adulthood. Socially isolated rodents exhibit changes in non-social behaviors such as heightened reactivity to new environments, impaired acoustic startle response, increased ethanol intake, and heightened anxiety. They also exhibit reduced social behaviors like play-fighting and grooming and increased aggression toward conspecifics [40]. Recent findings show that female rats exposed to social isolation exhibited impaired social preference and reduced social approach after weaning.

Furthermore, isolation led to decreased oxytocin activity in social contexts. These findings strongly suggest that post-weaning social experiences significantly influence sociability development in female rats. The implications of this study could be crucial for developing treatments for social dysfunction associated with neuropsychiatric disorders in humans [40].

The impact of post-weaning social deprivation on non-human primates, such as male guinea pigs, has been extensively studied. In their natural habitat, guinea pigs form enduring social bonds within heterosexual colonies. Post-weaning social deprivation involves isolating male guinea pigs from the colonies at 30 days after weaning but before sexual maturity and housing them individually or with a female until adulthood. This deprivation results in a lack of exposure to confrontations with older dominant males, which is crucial for developing appropriate social skills. Males reared alone or with a female exhibited difficulty forming stable dominance relationships and displayed increased aggression towards unknown conspecifics. These contrast sharply with colony-reared guinea pigs, who adapt quickly to new social situations, highlighting the importance of early social interactions for developing appropriate social skills. In non-human primates, establishing solid social skills through early interactions is essential for seamlessly integrating into stable social structures and approaching unfamiliar peers non-aggressively [27].

### 2.3. Limited Bedding and Nesting

Lastly, the limited bedding and nesting (LBN) paradigm offers another insightful model for studying the effects of ELS in rodents. In this model, the mother and her pups are placed on a wire mesh with only one-third of the standard nesting material and one-fourth of the bedding material from postnatal day 2 to day 9. These impoverished conditions prevent the dam from constructing an adequate nest, resulting in pups frequently falling out and experiencing fragmented maternal care, thus stimulating an abusive environment. Although the total maternal care time (including liking, grooming, and presence with pups) remains unchanged, the pups exhibit reduced body weight before weaning, indicating potential malnutrition. Comparing LBN-exposed pups to the control, LBN leads to several neurodevelopmental consequences, such as altered somatic development and body growth, higher basal plasma corticosterone levels, reduced corticotrophin-releasing hormone levels in the paraventricular nucleus of the hypothalamus, reduced hippocampal-expression of glucocorticoid receptors (GRs) and mineralocorticoid receptors (MRs), and short- and long-term memory deficits. Some of these effects are more evident in female offspring and manifest in adulthood rather than in adolescence, indicating that early-life adversity has a sex-dependent impact on cognitive and affective behaviors relevant to human psychopathology. Interestingly, exposure to LBN from P10 to P20 does not directly affect behavior but increases susceptibility to adult stressors, likely due to epigenetic and transcriptomic changes established during this period [3].

The lack of standardized protocols, such as variations in the type of stressor, its duration, and the age at which it is applied, presents significant challenges in comparing results across different studies and laboratories. These inconsistencies can impact the reproducibility and reliability of findings, underscoring the need for uniform methodologies used in ELS studies.

### 2.4. Limitations of Animal Models

Animal models of ELS provide important insights into the mechanisms behind early-life adversity. However, these models face significant limitations due to species differences, genetic and environmental variability, and the challenges of replicating human behavioral and psychological experiences. Standardizing these models is crucial to improving the validity and relevance of findings applicable to humans [41].

The interplay between genetic and environmental variation introduces significant challenges in accurately mimicking human conditions in animal studies and how they influence stress response. Since rodents are often inbred, findings from such homogenous animal populations may not directly translate to humans with diverse genetic backgrounds. Additionally, animal models are maintained in tightly controlled environments with minimal variations in diet, social interactions, and physical surroundings. Although some studies incorporate environmental enrichment to create more complex settings, these conditions still do not fully replicate the rich and diverse environments experienced by humans [42].

Behavioral tests in animals, such as the open-field test, the elevated maze, and the forced-swim test, utilized to measure aspects of behavior related to anxiety and depression may not fully recapitulate the range of human psychological experiences, especially given the complexity of human social interactions and cognitive processes. Human behavior is influenced by various cultural, social, and environmental factors that cannot be modeled in animals [43].

Moreover, it is crucial to recognize that the human brain’s intricate nature, prolonged development, and susceptibility to stress timing highlight the necessity for careful consideration when extrapolating findings from animal models to human scenarios. Thus, although animal models are invaluable for comprehending ELS, it is essential to acknowledge their limitations and exercise caution in applying their findings to human contexts.

## 3. Early-Life Stress in Humans and Its Effects

The effects of ELS can have lasting impacts on individuals, increasing their susceptibility to mental health disorders later in life. However, it is important to note that resilience, defined as the ability to adapt positively to adversity, is frequently observed. Factors such as the severity, type, duration, and timing of stressors and protective factors at both individual and environmental levels contribute to the variability in vulnerability and resilience to ELS [44].

Research on human models of ELS is essential to understanding the complex impacts of early adverse experiences on biological, psychological, and behavioral processes across a human’s lifespan. By unraveling these mechanisms, researchers aim to develop targeted interventions and preventive strategies to support resilience and improve long-term well-being in individuals affected by early adversity.

Prenatal stress in humans and other mammals refers to exposures during gestation that affect fetal development. Studies have shown that prenatal stress, ranging from severe to mild, can negatively influence pregnancy outcomes and the behavioral and psychological development of offspring. Various conceptualizations of prenatal stress in the human literature reflect the diversity of stressors experienced during gestation, including psychosocial stressors, such as changes in personal life, job status, housing, and domestic violence. These stressors require individuals to adopt adaptive coping behaviors. Consider, for example, the groundbreaking discovery by Caspi et al. (2002) [45], supported by other researchers [46,47], regarding the impact of polymorphisms in the promoter region of the MAOA gene. This gene encodes the monoamine oxidase A enzyme, which plays a crucial role in neurotransmitter breakdown in the brain. Their findings showed that the expression of this gene can influence antisocial behavior. They specifically found that individuals with the low-activity MAOA genotype are more susceptible to developing antisocial behavior when exposed to childhood maltreatment. In contrast, those with the high-activity genotype are less likely to exhibit antisocial behavior. These studies have substantially impacted the understanding of the biological mechanisms underlying the development of antisocial behavior and the role of gene-environment interactions [48].

Distress and anxiety during pregnancy can have profound effects on fetal and neonatal brain development, leading to long-term neurobehavioral dysfunction in children and adults. Maternal distress during pregnancy has been associated with structural and functional changes in the fetal brain, including alterations in hippocampal and cerebellar volumes, cortical gyrification, and functional connectivity [49]. Adverse neurodevelopmental outcomes, such as cognitive, language, learning, and memory; social–emotional problems; and neuropsychiatric dysfunction are increasingly observed in children exposed prenatally to maternal distress. Mechanisms implicated include impaired placental function, microbiome alterations, inflammation, fetal epigenetic changes, and maternal sleep and appetite disturbances [50].

Furthermore, women who have experienced childhood maltreatment (emotional, physical, sexual abuse, or neglect) face unique challenges during pregnancy and postpartum. Distress and mental health problems are common during pregnancy and postpartum, affecting up to 20% of women. Women with adverse childhood experiences are particularly at risk for postpartum depression, anxiety, and post-traumatic stress disorders. Maternal distress can perpetuate the cycle of maltreatment across generations, with maltreated mothers potentially more likely to have children who experience maltreatment and emotional and behavioral problems. Studies by Schury et al. (2017) have demonstrated that with the rising severity of childhood maltreatment, mothers experience higher levels of postnatal distress. Significantly, social support provided by friends, but not by the partner, parents, or parents-in-law, can buffer the adverse effects of childhood maltreatment on postnatal distress [51].

Social stressors, such as hierarchies and isolation, can induce lasting behavioral and physiological changes in individuals. Human-induced stressors, such as climate change and pollution, significantly impact biodiversity and global ecosystem functioning. While classifying stressors based on their origin and characteristics is valuable, it is essential to recognize their collective impact on biological systems.

## 4. Early-Life Stress and Its Effects on Molecular Pathways

ELS can impact crucial molecular pathways, such as the hypothalamic–pituitary–adrenal axis, epigenetic modifications, neurotransmitter systems, neurotrophic factors, and neuroinflammation.

### 4.1. Hypothalamic–Pituitary–Adrenal Axis

The impact of ELS on the HPA axis is a critical area that demands our attention and action. Evidence suggests that ELS could increase the risk of developing and sustaining severe mental health disorders in adulthood due to ongoing dysregulation within the HPA axis. The HPA axis plays a crucial role in the body’s response to stress, encompassing the paraventricular nucleus (PVN) of the hypothalamus, pituitary, and adrenal glands. In response to stress, the HPA axis triggers the well-known ‘fight-or-flight’ response [1]. The functioning of the hypothalamus within the HPA axis is influenced by various neurotransmitters, including excitatory ones like norepinephrine and serotonin, as well as inhibitory ones like γ-aminobutyric acid and opioids [52].

Corticotropin-releasing factor (CRF), a crucial regulator of the HPA axis, is produced in PVN neurons and released into the hypophyseal portal vessels. CRF travels to the pituitary gland and binds to CRF R1 receptors in the anterior pituitary corticotropes, prompting the synthesis and release of adrenocorticotropic hormone (ACTH). ACTH then travels through the bloodstream to the adrenal cortex, stimulating the synthesis and release of GCs [1]. This process is part of a feedback loop that regulates cortisol secretion in response to stress and maintains a physiological balance in the body. In humans, the primary GC is cortisol, while in rodents, it is predominantly corticosterone. The cellular and molecular actions of GCs begin upon binding to GRs. After steroid ligand binding, a conformational change occurs within the GRs, facilitating its translocation into the nucleus. Once in the nucleus, the GRs bind to specific DNA motifs known as glucocorticoid response elements (GREs) in the promoter region of glucocorticoid-responsive genes. This interaction with GREs regulates gene expression through its interaction with transcription factors [1].

GCs in circulation have widespread effects on various tissues and physiological processes in the body, including mobilizing energy reserves by increasing glucose production, promoting lipolysis (breakdown of fats) and proteolysis (breakdown of proteins), suppressing reproduction function, and influencing stress-related behaviors contributing to homeostasis [53]. Overall, stress responses, such as heightened alertness and metabolism, along with the suppression of immune function, effectively improve the body’s response to stressors by providing energy and enhancing cognitive function to deal with immediate challenges.

However, chronic HPA activation, leading to sustained elevation of GCs, can harm various physiological systems. Prolonged exposure to elevated GC levels has negatively impacted immune function, cardiovascular health, metabolic regulation, and neural function. Indeed, this chronic activation may reduce the resilience of neurons and glial cells to subsequent challenges [54], making them more vulnerable to subsequent challenges and thereby increasing their susceptibility to various diseases.

Recent literature has described how the HPA axis undergoes careful regulation throughout life. Early life events can alter the responsiveness of the HPA axis permanently. The mother’s presence during early life is crucial for pups, as it reduces anxiety-like behavior and prevents short-term memory impairment later in life. This nurturing presence protects against the adverse effects of early-life social stress [55]. Some studies suggest that the neonatal period, around birth, is a sensitive stage of life when active neuroplasticity occurs in the developing brain, and stress can impact it through various mechanisms. In mice, the period from postnatal days (PNDs) 1 to 12, and in rats, PNDs 3 to 14, is known as the stress hyporesponsive period (SHRP) and is considered a critical developmental phase. During this time, the adrenal glands exhibit reduced sensitivity to ACTH and most stressors. Adequate maternal care also reduces the secretion of ACTH and corticosterone in pups [21]. As a result, brain development progresses optimally in developing mouse and rat pups, with the SHRP potentially serving as a protective phase for the developing brain [21,55].

Numerous studies have confirmed the negative impact of early separation from the mother or MD. MS, ranging from 1 to several hours, is stressful for neonate animals, mainly if it occurs repeatedly. This separation significantly alters ACTH and corticosterone levels [33]. Furthermore, MS significantly alters individual processes of postnatal neurogenesis in the olfactory neurogenic region—the subventricular zone (SVZ) and the rostral migratory stream (RMS). Fabianova et al., 2018 demonstrate that single exposure to MS stimulates cellular activity in the SVZ and anterior olfactory nucleus [56]. Numerous studies have shown that MD impacts various behaviors and leads to the long-lasting dysregulation of the HPA axis in different species [57,58]. In rodents, the nature and timing of exposure to MD play a critical role in neurodevelopmental disorders [59]. Similar consequences of reduced maternal care have been observed in monkeys and humans [31,60,61]. A recent article describes the harmful effects of MD on rhesus monkeys, resulting in structural abnormalities in the visual cortex and premature myelination in the posterior superior temporal sulcus [62]. In humans, the most severe early-childhood stressful events typically involve deprivation, disruption, neglect, or abuse [63,64].

Maternal deprivation in young mice has been found to increase corticotropin-releasing hormone (CRH) mRNA expression in the parvocellular neurons in the PVN. This upregulation, in turn, heightens their excitatory input, resulting in exaggerated ACTH and corticosterone responses [65]. Furthermore, MD reduces the expression of glucocorticoids and MRs in the hippocampus due to disrupted negative glucocorticoid feedback [65].

#### Developmental Changes in the HPA Axis: Pre-Puberty to Late Puberty

The HPA axis is generally more reactive to stress in the pre-pubertal stage. Basal and stress-induced levels of glucocorticoids tend to be lower than in adulthood, but the response to acute stress is often more pronounced. The regulatory mechanisms of the HPA axis, including negative feedback by glucocorticoids on the hypothalamus and pituitary, are less efficient in younger individuals, resulting in a prolonged elevation of glucocorticoid levels following stress [66]. ELS can lead to a blunted glucocorticoid response in the pre-pubertal stage, likely due to alterations in the sensitivity of glucocorticoid receptors, changes in the expression of HPA axis regulatory genes, and epigenetic modifications. Brydges et al. (2020) explored how the HPA axis in female rats reacts more sensitively to stress experienced before puberty. Females exposed to pre-pubertal stress showed increased susceptibility to stress-related disorders such as anxiety and depression. This increased sensitivity is believed to have significant implications for understanding sex differences in the development of stress-related disorders, highlights the importance of considering sex differences in research on stress, and underscores the need for early interventions to mitigate the long-term effects of pre-pubertal stress, especially in females [67].

Puberty is a critical period of maturation for neuroendocrine systems that brings significant hormonal changes, including increases in sex steroids (e.g., testosterone, estrogen), which interact with the HPA axis. These changes can modulate stress reactivity and glucocorticoid levels. The HPA axis shows considerable plasticity during puberty, and exposure to adverse experiences during puberty can impede normal brain reorganizing and remodeling and result in enduring consequences on brain functioning and behavior [1].

Kolmogorova and Ismail (2021) demonstrated that during puberty, LPS administration increases the levels of hippocampal PSD95, a crucial protein linked to learning and memory, in male mice but not in female mice at one-week post-treatment. This finding suggests that activating the HPA axis and immune system may have a sex-specific influence on the expression of essential molecules in brain regions that directly impact learning and memory [68]. Furthermore, female pubertal rats exposed to social instability exhibit decreased spatial and object memory in adulthood. In addition, these rats demonstrate lower hippocampal cell proliferation and survival levels compared to their non-stressed counterparts [69].

In humans, research suggests that exposure to stress during puberty is associated with 33% of depression and 16% of anxiety disorder cases [70]. Human females are two times more susceptible to developing depression than males. Murray et al. (2019) demonstrated that LPS treatment leads to increased anxiety-like behavior in male rodents and increased depression-like behavior in female rodents in a long-lasting manner [71]. Similarly, exposure to predator stress during puberty led to increased anxiety symptoms in both adult male and female mice [72]. The study by Murack et al. (2021) found that experiencing chronic sleep deprivation for seven days during puberty can also lead to increased depression-like symptoms in adult males and females. In females, sleep deprivation leads to a reduced expression of serotonin 1A receptors in the CA2 region of the hippocampus, the cingulate cortex, and mPFC [73].

By the end of puberty, the HPA axis stabilizes. Basal glucocorticoid levels rise compared to pre-pubertal stages, and the acute stress response becomes finely regulated. After puberty, sex differences in HPA axis function become evident: females generally have higher basal cortisol levels and a more robust stress response than males. Post-puberty or adult individuals exposed to ELS often develop a hyper-reactive HPA axis, leading to higher basal glucocorticoid levels and an exaggerated stress response [21]. Dysregulation of the HPA axis due to ELS is strongly linked to aggression, maladaptive behaviors, and a higher incidence of mental health disorders in adulthood. Conditions such as depression, anxiety, PTSD, and other mood disorders are more prevalent among individuals with a history of ELS [21]. The altered stress response can worsen symptoms and complicate treatment.

ELS profoundly impacts the HPA axis and various molecular pathways (see below neuroinflammation, epigenetic modifications, neurotrophic factors, synaptic plasticity), leading to long-term changes in brain structure and function. As we discuss in the following sections, these changes can make individuals more susceptible to mental disorders and neurodegenerative diseases, emphasizing the importance of early interventions to reduce the adverse effects of ELS.

### 4.2. Early-Life Stress and Epigenetic Modifications

ELS induces long-term phenotypic adaptations that increase vulnerability to neuropsychiatric disorders through epigenetic mechanisms, including DNA methylation, histone modifications, and noncoding RNA activity [23].

ELS shapes epigenetic profiles in systems like the HPA axis, monoamine, and neuropeptides, leading to differential methylation in gene promoters [74]. For instance, maternal care influences offspring DNA methylation patterns, notably at the GR promoter, affecting stress responses. Methylation changes also impact synaptic plasticity in the hippocampus by modulating neurotrophic factors. One mechanism involves exon IV methylation influencing brain-derived neurotrophic factor (*Bdnf*) gene expression, which is susceptible to environmental factors. Studies show that early maltreatment increases *Bdnf* DNA methylation at exons IV and IX in the prefrontal cortex, while decreased methylation is observed in the hippocampus following exposure to predator odor [75].

As reviewed by Zhang et al. (2013) [76], there is a profound connection between ELS and the epigenetic landscape, mainly focusing on the methylation of the Crf gene promoter. This methylation correlates with CRF mRNA levels in the central amygdala during learned helplessness. Prenatal stress can alter the methylation of genes that encode adhesion molecules and neurotransmitter receptors, leading to decreased expression of glutamatergic receptors (GluR1) and glutamate transporters (glial EAAT2 and neuronal EAAT3) in key brain regions such as the hippocampus, frontal cortex, and striatum. In rats, prenatal stress affects the methylation of the neuronal membrane glycoprotein gene (gpm6a) and impairs hippocampal neurogenesis.

Maternal behaviors, particularly licking and grooming (LG), are crucial in shaping these epigenetic modifications. Offspring of high LG mothers show CpG hypomethylation, which increases hippocampal GR expression and reduces stress responses. Conversely, low LG mothers’ offspring show CpG hypermethylation. Detailed mapping of the exon 1 reveals significant methylation differences at specific CpG sites in the hippocampus. The 5′ CpG site of the NGFI-A sequence is hypermethylated in low LG offspring and hypomethylated in high LG offspring. Cross-fostering experiments have demonstrated that these methylation differences can be reversed, underscoring the direct impact of maternal care on DNA methylation [76].

McGowan et al. (2009) found that low licking and grooming behavior leads to differential NR3C1 promoter methylation, which affects hippocampal GR expression and HPA stress responses [77]. ELS causes sustained DNA hypomethylation in the arginine vasopressin gene regulatory region in mice. In humans, childhood maltreatment is linked to decreased hippocampal GR expression and increased stress responses later in life, aligning with findings from animal studies.

The study of epigenetics in the context of ELS and psychiatric illness during adolescence is an emerging area of research. In C57BL/6 mice, a two-hit stress model involving early-life MS combined with adolescent social isolation reveals sex-specific anxiety and depression phenotypes, implicating epigenetic changes in genes such as Nr3c1 and Cacna1c in these behavioral alterations [78].

Yang et al. (2012) and Fitzgerald et al. (2021) explored stress’s impact on DNA methylation in mice, highlighting the significance of epigenetic modifications in response to early-life stressors. Yang demonstrated a stress-induced reduction in FKBP5 intron 5 in males, pointing to specific gene regulation changes [79]. Complementary, Fitzgerald et al. (2021) provided a broader perspective by showing extensive DNA methylation changes in the hypothalamus of young mice associated with behavioral outcomes like hyperactivity in adulthood, thereby underscoring the potential long-term effects of ELS on brain function and behavior [80].

In 2023, Demaili et al. explored the effects of ELS and adolescent stress on regulatory elements of the endocannabinoid system in the medial prefrontal cortex of adult female rats. They focused on the cannabinoid receptor 1 (CB1R) and fatty acid amide hydrolase (FAAH). They examined whether these effects involved changes in DNA methylation. They found that ELS and forced swimming (FS) during adolescence upregulated CB1R gene expression, while FAAH gene expression was upregulated only in the FS group. ELS, occurring during a critical stage of cortical development, might permanently alter gene expression, modulating responses to FS. They also found that increased gene expression was associated with reduced DNA methylation in the promoter region. This implies that ELS influences long-term programming by altering how genes respond to stress during adolescence through specific epigenetic modifications [81].

In 2020, Catale et al. [82] emphasized the diverse impacts of different types of ELS on epigenetic programming. Through experiments with adult mice, he demonstrated how ELS experiences linked to depression-like (ESI) or addiction-like (ESS) behavior had distinct effects on subcortical brain regions involved in emotion, reward, and cognitive processes. Notably, the study revealed a significant increase in global DNA methylation levels in the striatum of ESI mice compared to both control and ESS mice. Similarly, the hippocampus showed a marked elevation in global DNA methylation levels in ESI mice compared to ESS mice.

These studies collectively highlight that ELS induces pervasive epigenetic modifications during adolescence, closely linked to behavioral responses. However, variability in animal models and strains necessitates further replication studies to ensure robustness and reliability. Moreover, investigating region-specific and cell-type-specific epigenetic changes will deepen our understanding of ELS’s impacts on brain development and function.

Importantly, the potential reversibility of epigenetic states in the adult brain presents exciting opportunities for pharmacological interventions. Histone deacetylases (HDACs), crucial for removing acetyl groups, play a significant role in this context, offering promising therapeutic avenues to mitigate the adverse effects of ELS.

### 4.3. Neurotrophins Modulation and Neurodevelopment

The intricate interplay between genetics and environment profoundly influences brain development, especially during the critical early postnatal period. It is fascinating how experiences can shape the growth of neural connections, from the initial stage of neurogenesis to the refinement of synapses, under the modulatory effects of neurotrophins like nerve growth factor (NGF) and BDNF.

Neurotrophins like NGF and BDNF serve as master regulators of brain development, orchestrating essential processes, such as neuronal proliferation, survival, and differentiation, as well as synaptic plasticity across the central and peripheral nervous systems [83,84,85]. However, ELS can disrupt this delicate equilibrium by altering the expression of essential neurotrophins through epigenetic mechanisms, like DNA methylation. This disruption can derail the intricate neural programming that unfolds during early development, potentially leading to a cascade of long-term consequences on brain maturation and increased vulnerability to neuropsychiatric disorders and neurodegenerative diseases [83,86].

Fascinatingly, studies on rats subjected to early-life MS have shown increased NGF expression in the hypothalamus, cerebral cortex, and hippocampus and increased cell death in specific brain regions [87,88].

Additionally, research by Rocheri et al. (2022) demonstrated that adult rats deprived of maternal care during postnatal life exhibited reduced expression of BDNF and NMDA receptors in the hippocampus [89]. Moreover, a recent work by Tran et al. (2022) highlighted the complex effects of MS in adult (PND98) rat brain, inducing the expression of six variants of *Bdnf* mRNAs. In the VTA and substantia nigra, MS upregulates Bdnf IIA and III mRNA levels more than in controls. By contrast, in the ventral striatum, Bdnf I, IIA, IIC, IV, and VI transcripts had significantly lower expression in maternally separated rodents than in the control. In addition, Bdnf I and IIC mRNA expression increases more in the midbrain of female rats exposed to stress than in males.

ELS can have profound and enduring effects on brain regions involved in DA neurotransmission, fundamentally altering the trophic environment and potentially reshaping responsiveness to subsequent stressful events in a sex-specific pattern [90].

Maternal deprivation in early life leads to significant brain remodeling, resulting in abnormal maturation or rewiring of neuronal connectivity [91]. Studies have revealed increased excitatory synapses in the murine hypothalamus in response to stress [65]. However, chronic ELS in rodents and humans has been associated with reduced dendritic complexity, altered synaptic number and function, and overall structural changes in brain regions crucial for emotion regulation and memory processing, such as the hippocampus, PFC, and amygdala [92,93]. These findings underscore the significance of early life experiences on brain development and emphasize the need to create a supportive environment for optimal brain maturation.

There is growing evidence that environmental interventions can mitigate the long-term consequences of ELS on brain development. Research by Menezes et al. (2020) indicates that administering environmental enrichment to rat pups post-weaning increased hippocampal BDNF levels and protected against cognitive deficits [94]. Moreover, an enriched environment has been found to have an impact on alcohol intake and aggressive behaviors in rats, which are often linked to ELS [95]. Environmental enrichment has also shown effectiveness in reversing anxiety- and depression-like behaviors caused by ELS [96]. Indeed, Papadakakis et al. (2019) reported that music, as a form of environmental enrichment, improved the behavioral impairments caused by ELS in rats [97]. In addition, as revised by Speranza et al. (2022), music can influence the release of neurotransmitters (DA, serotonin, and oxytocin) and hormones, ultimately affecting the reward and prosocial systems. Music is also used to develop therapies for neurological disorders [98]. These findings align with studies on early-life parent–child separation, suggesting that an enhanced and stimulating adolescent environment can recalibrate human cortisol reactivity [99,100]. New research confirms that exposure to ELS generally reduces neurotrophin levels in rodents. However, aerobic exercise has the opposite effect, increasing neurotrophin expression and potentially offering neuroprotective and neurogenic benefits [86].

The interconnectedness of ELS, environmental enrichment, brain chemistry, and therapeutic interventions underscores the importance of holistic approaches.

### 4.4. Neurotransmitter Systems

ELS can have profound and lasting effects on various neurotransmitter systems in the brain, including serotonergic, GABAergic, and dopaminergic systems. These systems play crucial roles in regulating mood, emotion, reward, and stress responses [101,102]. Here, we briefly summarize the impact of ELS on the serotonergic and GABAergic systems while thoroughly investigating its effect on the dopaminergic system.

#### 4.4.1. Impact of Early-Life Stress on Serotonergic and GABAergic Neurocircuitry

ELS can profoundly affect serotonergic neurocircuitry through multiple mechanisms, including alterations in serotonin synthesis [103], receptor function [104,105,106], transporter expression [107,108], and innervation of key brain regions [109]. These changes can contribute to HPA axis dysregulation, leading to heightened stress reactivity and increased susceptibility to psychiatric disorders [103].

Similarly, ELS significantly impacts the GABAergic system, which is essential for maintaining the balance between neural excitation and inhibition. This system regulates anxiety, stress responses, and overall brain function. ELS disrupts GABA synthesis, receptor function [110,111], and neurosteroid modulation [112]. It also impairs the inhibitory control of the HPA axis, leading to increased sensitivity to stressors [113]. Consequently, ELS exerts an inhibitory effect on key brain regions responsible for emotion regulation, stress responses, and cognitive function, thereby increasing vulnerability to anxiety, stress-related disorders, and other psychiatric conditions [114].

#### 4.4.2. Impact of Early-Life Stress on Dopaminergic Neurocircuitry

A robust body of research has shown that ELS can significantly impact mental health by affecting dopaminergic neurocircuitry. This circuitry is crucial for reward processing, cognitive flexibility, and goal-directed behavior. Disruptions in this circuitry have been linked to symptoms such as anhedonia, impulsivity, and reduced motivation, which are common factors in adult psychopathology [110]. It is important to note that there is limited understanding of how stressful experiences affect dopaminergic circuitry in early childhood. This is a critical time for comprehending how stress leads to long-term biological changes.

Research on animal models has compellingly illustrated that ELS has the potential to induce significant alterations in the DA system. These changes impact excitability, receptor number and sensitivity, DA reuptake, and metabolism [115].

The ventral tegmental area (VTA) in the midbrain has been identified as a crucial target for studying the impact of stress on the reward circuitry [116,117]. The VTA serves as the primary source of DA projections to other reward-related regions, such as the nucleus accumbens (NAc), anterior hippocampus, and medial PFC, collectively forming the mesocorticolimbic pathway [102,118,119].

Recent studies using rodent models have indicated that ELS may generate long-term effects in the VTA, including alterations in the expression of Otx2, a transcription factor critical for dopamine neuron development and plasticity. These changes can make individuals more vulnerable to later stress and depression-like behaviors [120]. In 2019, Peña et al. also demonstrated that ELS during a postnatal sensitive period could alter brain circuit development and response to later stress and rewards, thereby increasing susceptibility to psychiatric disorders [121,122]. Specifically, they found that ELS during postnatal days P10–P20 increases sensitivity to adult stress in female and male mice, evidenced by increased depression-like behavior following additional stress in adulthood. These effects are likely mediated by transcriptional alterations in distinct genes potentially involved in common signaling pathways related to neurite outgrowth and synapse formation, observed in male and female mice across three brain reward regions: the VTA, NAc, and PFC [121]. Complementary work suggests that stress exposure in early adolescent rats results in abnormal VTA and anterior hippocampus activity, potentially leading to enduring alterations in the VTA and latent vulnerability for dysregulated reward-related behaviors [123].

ELS also induces the reactivation of ELS-sensitive cellular ensembles in mesocorticolimbic regions of the brain. In a study by Balouek et al. (2023), the authors used transgenic mice (ArcCreER^T2^) to permanently label neurons activated by early experiences and monitor their dynamics throughout the lifespan. They found that neuronal ensembles in key mesocorticolimbic regions remain hypersensitive to stress across the lifespan. Additionally, when chemogenetic techniques were used to inhibit these ELS-activated neurons in the NAc, there was a reduction in stress hypersensitivity in adult male mice, an effect not seen in female mice, indicating a sex-specific difference in response to ELS [124].

Further research in rodents shows that chronic stress can lead to the loss of dopamine neurons in the VTA and functional changes in its connections with other brain regions, impacting how the mPFC regulates the VTA. This indicates a prolonged period during which VTA dopamine neurons are susceptible to environmental stress. In humans, ELS is linked to changes in the activation and connectivity of reward-processing regions, as demonstrated by FMRI studies focusing on the NAcc and mPFC [125,126,127,128,129]. For instance, childhood maltreatment affects NAcc–mPFC connectivity, and additional stress can worsen these effects [127]. ELS also influences VTA–hippocampus connectivity in late childhood and adolescence, though its impact during early childhood is less understood [130]. A study by Park et al. (2021) explored how ELS, defined by socioeconomic status (SES) and Adverse Childhood Experiences (ACEs), affects VTA connectivity development from ages 4 to 9. It also examined the connectivity of other mesocorticolimbic regions (NAcc, aHipp, and mPFC) to see if VTA-related changes are specific and how connectivity patterns change with age based on stress exposure [131].

Animal studies show that ELS profoundly affects the VTA, which releases dopamine in regions like the NAcc, anterior hippocampus, and mPFC, causing cascading developmental effects. These findings align with research by Pena et al. (2019), indicating that ELS causes changes in the VTA, making individuals more susceptible to later stressors [121].

Evidence suggests that chronic stress results in the loss of dopaminergic neurons in the VTA and changes in dopamine activity in its target areas, such as NAcc and mPFC. Stress also alters mPFC neuron morphology. The communication between VTA and mPFC is complex and bidirectional, with excitatory and inhibitory connections influencing dopamine activity. Animal models have not yet determined whether ELS affects projections from the VTA or to the VTA from the mPFC. Unfortunately, it cannot distinguish between excitatory and inhibitory connections, and more data are needed to understand directionality in young children. This study supports cross-species evidence that VTA projection development in rodents and dopamine availability maturation in humans continue through childhood and adolescence. A blunted VTA–mPFC developmental trajectory might indicate reduced resilience to stress, putting children at higher risk for developing psychopathology after later life stressors. To counteract the long-lasting effects of early disruptions to reward neurocircuitry, it is crucial to design early-childhood interventions that help children develop effective stress-coping strategies and promote positive, motivated behavior [131].

Disruptions to the brain’s reward circuitry and pathways responsible for motivation and emotion have been linked to the effects of early-life adversity (ELA) on adult behavior. The specific molecular mechanisms behind the long-term impact of ELA remain unclear. Hamdan et al. (2022) [132] investigated this issue using neonatal MS as a form of ELA to see if it may alter behavior and synaptic protein expression in adult rats. In their study, male rats were separated for 180 min daily during postnatal days (PND) 2–14 and then allowed to grow to adulthood (PND 60) without further manipulation. The researchers hypothesized that this separation would impact dopaminergic and glutamatergic signaling and increase sensitivity to methamphetamine. They analyzed brain regions (Hipp, mPFC, NAc, and CPu) for various proteins related to dopamine neurotransmissions. The findings revealed that rats subjected to MS exhibited increased anxiety-like behavior compared to control rats. They also showed conditioned place preference (CPP) to methamphetamine, although their responses did not significantly differ from controls at any dose tested. Increased expression of NMDA receptor (NMDAR), dopamine receptor-2 (D2), and α-synuclein (ALPHA) was observed in the NAc and CPu.

Additionally, elevated levels of D2, ALPHA, and dopamine transporter (DAT) were found in the mPFC, alongside reduced expression of dopamine receptor-1 (D1). The hippocampus showed reduced expression of both D1 and D2, while the CPu had elevated levels of tyrosine hydroxylase (TH) and decreased DAT expression. No significant changes were observed in postsynaptic density 95 (PSD95) expression in MS rats. These findings suggest that ELA leads to region-specific and long-lasting changes in synaptic protein expression, which reduce dopamine neurotransmission and increase anxiety-like behavior in adult rats. Such alterations may contribute to the heightened vulnerability to stress-related illness seen in individuals with a history of early-life adversity [132].

The lateral habenula (LHb) is a brain region involved in decision-making and stress response, influencing dopamine-related reward pathways. Increased LHb activity is linked to addiction, schizophrenia, and stress disorders. The study conducted by Simmons et al. explored how Dynorphin/Kappa opioid receptor (Dyn/KOR) signaling affects LHb function, especially after ELS, such MD. Using rat brain slices, researchers found KOR activation impacts LHb neuron subtypes differently, altering their excitability and synaptic transmission. MD increased Dyn levels and decreased KOR expression in the LHb, disrupting normal KOR-mediated neuronal responses. This study highlights the role of Dyn/KOR signaling in LHb and its modulation by ELS [133].

ELS can also have a significant impact on the susceptibility to addiction in adulthood. Research has shown that ELS is a considerable risk factor for alcohol, morphine, methamphetamine, cocaine, and cannabinoid abuse in adulthood [134]. This influence is likely due to the modulation of the mesolimbic dopaminergic reward pathway. Studies have indicated that there is a strong connection between stress and substance abuse, involving neurotransmitter systems such as the opioid and DA systems [135,136]. Research has shown that continuous MS during the first days after birth can result in long-lasting changes in the concentration of DA receptors in the brain, as well as increased uptake of ethanol, cocaine, and morphine in adolescent and adult male rats [135,137,138]. Ploj et al. (2003) [135] discovered that MS inhibits the expression of D2 receptors in the VTA and frontoparietal cortex. This inhibition suppresses the negative feedback regulation of DA in the same brain areas, ultimately enhancing DA activity in these regions. The link between ELS and adult drug abuse is also supported by neurochemical changes in the brain’s serotonin system [139], alterations in the HPA axis [140], and changes in the endocannabinoid system [141,142,143]. Studies showed that neonatal isolation from the mother is associated with increased cocaine abuse and greater sensitivity to cocaine in both animals and humans [144,145,146]. Maternal deprivation leads to altered endocannabinoid system function in adulthood and increases the risk for impulsive and depressive-like behavior, which in turn heightens the risk of cannabinoid abuse [147,148]. Similarly, individuals who experienced childhood abuse or neglect are at a higher risk for substance abuse later in life, likely due to similar alterations in the dopaminergic system, as observed in animal studies [149,150,151].

Understanding the impact of ELS on the development, function, and motivated behaviors of the reward circuit is crucial. By unraveling these complexities, one can foresee, prevent, and treat stress-related mental health conditions, including mood, anxiety, and substance use disorders.

#### 4.4.3. Sex-Specific Responses to Early-Life Stress

Sex-specific differences in stress responses are influenced by various factors, including hormones from the hypothalamic–pituitary–gonadal (HPG) axis. Two critical hormones in this axis are estrogen and testosterone, which significantly affect the body’s stress response system, including the HPA axis.

Research has shown sex differences in how individuals experience and respond to ELS. Women have greater vulnerability to the effects of ELS, characterized by a 2:1 female/male risk ratio for stress-related pathology. This increased susceptibility in women influences the prevalence, symptoms, onset, course, and treatment of various illnesses, though the underlying mechanisms remain poorly understood.

Recent rodent studies have identified sex differences, including genetic, hormonal, and gender-related aspects, which significantly influence responses to ELS and subsequent mental health outcomes. However, few animal models reliably replicate these findings in behavioral outcomes. For example, Goodwill et al. (2018) found that fragmented maternal care led to depressive-like symptoms in female mice, which were responsive to ketamine treatment [152]. This was observed using continuous home cage video monitoring, which revealed a female-specific depressive-like phenotype following ELS. Additionally, this approach demonstrated ketamine’s rapid antidepressant effects in females, mirroring responses observed in humans [152,153].

Genome-wide studies have revealed that ELS induces latent sex-specific transcriptomic changes, influencing susceptibility to neuropsychiatric disorders. Understanding these interactions holds promise for developing personalized therapeutic strategies [154].

Recently, research has illuminated the intricate interaction between the HPA and HPG axes, revealing distinct stress responses in males and females. This interaction is a critical factor in why women are diagnosed with mood disorders, such as depression, at approximately twice the rate of men. This discrepancy is heavily influenced by hormonal fluctuations, including those related to age and changes in hormonal states.

Estrogen, the primary female sex hormone, generally increases baseline levels of cortisol, an essential stress hormone. This elevation impacts how the brain regulates emotions and processes rewards. High estrogen levels during the follicular phase of the menstrual cycle may reduce the HPA axis response to stress, suggesting a protective effect. Estrogen receptors in brain regions such as the hippocampus, amygdala, and prefrontal cortex enhance neuroplasticity and promote neurogenesis in the hippocampus, which is crucial for resilience to stress. However, during periods of low estrogen, like the luteal phase or menopause, women often show a more pronounced HPA axis response to stress [155].

Testosterone, the primary male sex hormone, tends to decrease cortisol levels, influencing how stress and emotional responses are controlled [156].

Testosterone reduces the activity of the HPA axis by lowering the secretion of CRH and ACTH, resulting in decreased cortisol levels. This contributes to a less intense stress response in males compared to females. Testosterone also influences brain regions such as the amygdala, modulating behaviors related to aggression and social interaction. The higher levels of testosterone in males typically contribute to lower anxiety and a more controlled stress response.

The interplay between testosterone and other hormones, like cortisol, can further dampen male stress response. The impact of these hormonal interactions on depression-like behaviors in animal models has yet to be fully explored. For instance, in one study using a rodent model, researchers examined whether removing gonads or supplementing with testosterone affects depression-like behaviors. They focused on the PFC, a brain region important for emotion regulation and stress responses, to understand how gene expression related to the HPA and HPG axes is influenced [157,158].

During puberty, the surge in sex hormones leads to the development of sex-specific stress responses. ELS can interfere with this development, potentially causing long-term alterations in HPA axis function. The androgen hypothesis suggests a link between testosterone and suicidal behavior in men, though conclusive evidence is still lacking. Daily fluctuations in testosterone and cortisol levels add a layer of complexity to research and analysis. Changes in the androgen receptor gene could lead to depression in men, but more clinical evidence is needed [159].

Understanding how estrogen and testosterone affect stress responses can help tailor treatments for stress-related disorders. For instance, hormone replacement therapy in menopausal women or anti-androgen therapy in conditions with excessive testosterone might be adjusted to mitigate stress-related symptoms. As we delve deeper into these hormonal mechanisms, we can enhance our understanding and improve interventions for stress-related disorders across sexes.

### 4.5. Early-Life Stress and Immune Response

Experiencing ELS can weaken the body’s natural defense system against pathogens by affecting innate immune cells such as natural killer cells, neutrophils, and macrophages. ELS can also impact the development and function of adaptive immune cells like T and B lymphocytes, reducing the ability to mount effective immune responses and produce antibodies [160].

#### 4.5.1. Microglia Activation

ELS can initiate the activation of microglia, the immune cells in the CNS that are vital for neuroinflammatory responses. This activation involves the translocation of NF-κB (nuclear factor kappa-light-chain-enhancer of activated B cells) from the cytoplasm to the nucleus, where it binds to specific DNA sequences, promoting the transcription of various pro-inflammatory cytokines such as interleukin-1β (IL-1β), interleukin-6 (IL-6), and tumor necrosis factor-alpha (TNF-α), as well as reactive oxygen species (ROS) [161]. This sustained activation can contribute to chronic neuroinflammation, which may lead to persistent activation of the HPA axis and elevated cortisol levels. The latter can interact with immune cells in the brain, influencing their activation state and exacerbating neuroinflammatory responses [162]. The chronic neuroinflammatory state can damage neurons and other CNS cells, contributing to neurodegenerative processes [161]. Activated microglia exhibit morphological changes, becoming more amoeboid in shape, which indicates a transition to a more reactive state. This activation can persist into adulthood, suggesting long-term alterations in microglial function and brain immune responses. Delpech et al. (2016) have demonstrated that ELS perturbs the maturation of microglia in the developing hippocampus. Particularly, MS significantly impacts the density and morphology of microglia in the hippocampus of 14-day-old pups, with effects no longer present on postnatal day (PND) 28 [163].

In contrast, microglia at PND28 exhibited increased phagocytic activity and reduced expression of genes that typically increase across development [163]. Abnormal phagocytic activity was also linked with increased spine density in CA1 pyramidal neurons observed in 17-day-old groups and persisted in PND29 exposed to limited bedding and unpredictable postnatal stress mice [164]. In parallel, as demonstrated by Catale et al. (2020) [82], two different types of ELS experiences, ESI and ESS, impact the striatum, nucleus accumbens, amygdala, and hippocampus differently, not only on the epigenetic modifications but also on microglia. Microglia in ESS mice showed significantly enlarged soma in the striatum compared with control mice, and the IBA1^+^ cell numbers increased dramatically in ESI mice compared with control mice in the CA1 and CA3 regions. IBA1^+^ cell numbers were also considerably increased in ESS mice compared with control mice in the CA3 region. However, ESS mice had lower numbers of IBA1^+^ cells than ESI and control mice in the basolateral amygdala. In a recent study, Catale et al. (2022) [165] showed that exposure to social stress during the juvenile period in mice leads to the activation of microglia and abnormal self-inhibitory responses also in the developing VTA dopaminergic neurons, along with increased sensitivity to the effects of cocaine later in life. Treating with minocycline, a microglia activation inhibitor, or with GW2580, a selective inhibitor of macroglia/microglia functionality during social stress, normalized microglia and dopamine sensitivity in the VTA and prevented the development of susceptibility to cocaine [165].

The effects of ELS on microglia are complex and multifaceted, involving changes in activation state, cytokine production, synaptic pruning, epigenetic regulation, and regional specificity. These alterations, often mediated through the NF-κB pathway, can have long-lasting impacts on brain development and function, potentially leading to various cognitive and behavioral consequences. Understanding these mechanisms is crucial for developing interventions to mitigate the adverse effects of ELS on brain health.

#### 4.5.2. Early-Life Stress and Astrocytes Activation

ELS can have profound and lasting effects on the brain, including activating astrocytes, a type of glial cell involved in maintaining brain homeostasis. Astrocytes interact with neurons in multiple ways as part of the tripartite synapse. They affect neurotransmitter uptake and metabolism, provide gliotransmitters, and supply energy to neurons within local circuits. Activated NF-κB in astrocytes produces pro-inflammatory cytokines such as IL-1β, TNF-α, and IL-6. Inflammatory cytokines from activated astrocytes can impair synaptic function and plasticity. This disruption affects cognitive processes and neuronal communication [161]. Çaliskan et al. (2020) [166] discussed how astrocytes influence synaptic plasticity and how their functions and markers are affected by stress at different life stages. Most revised studies have focused on astrocyte markers like GFAP and S100ß in brain regions involved in emotional behavior, but only a few have examined specific astrocyte functions. Maternal separation for 3 h/day between PND 2 and 15 induces downregulation of GFAP expression in the PFC of PND 70 mice [167]. Other groups found similar results in the PFC, striatum, nucleus accumbens, and dorsal hippocampus [168,169].

Furthermore, ELS impairs astrocytic glutamate regulation, exacerbating excitotoxicity and elevating oxidative stress, overwhelming the astrocytes’ antioxidant defenses. The increased synaptic and tonic glutamatergic transmission leads to enhanced excitation of CRF-releasing neurons [170]. These alterations compromise the blood–brain barrier, increasing the brain’s vulnerability to neurotoxic substances. It has been demonstrated that MS increased the neuroinflammatory response to lipopolysaccharide (LPS) challenge in male rats, particularly in juveniles. In contrast, female rats exposed to MS exhibited no difference or a reduced central response to LPS compared to control females, especially in adulthood. These findings indicate that ELS may prime individuals to respond differently to environmental factors later in life in a sex-specific manner, potentially influencing their vulnerability or resilience to mental and neurodegenerative diseases [171].

Understanding the molecular mechanisms of how ELS affects astrocytes could help us pinpoint potential drug targets to reduce stress-induced changes in astrocytes. This, in turn, could open up new treatment options for stress-related mental health conditions like anxiety disorders and depression, which could be further explored in practical applications.

## 5. Impact of Early-Life Stress on Mental Health

ELS has profound and lasting effects on psychological health, increasing the susceptibility to various psychiatric disorders, such as depression, anxiety, and suicidal behavior, particularly after following further stressful events later in life [172]. Extensive research in human and animal models indicates that ELS sensitizes individuals to respond more intensely to future stressors. However, the cellular mechanisms linking early adverse experiences to heightened stress sensitivity and increased risk of psychiatric conditions remain elusive [124].

ELS significantly contributes to the increased probability of developing major depressive disorder (MDD), the most common psychiatric disorder [173]. A meta-analysis study reported an odds ratio (OR) of 2.1 (95% CI: 1.8–2.5) for MDD among those with ELS compared to those without [174]. The relationship between ELS and MDD involves many molecular mechanisms previously described, such as the activation of the HPA axis, the dopaminergic system, and the neurotrophin system. It also includes the serotonergic and oxytocin systems [175].

Shin et al. (2023) demonstrated that a novel ELS model, combining MS, limited bedding, and mesh platform conditions, induced anxiety- and depression-like behaviors, as well as social deficit and memory impairment in offspring mice [176].

Moreover, ELS is a significant risk factor for schizophrenia, a devastating disease influenced by genetic predisposition and environmental factors, such as urbanicity, obstetric complications, and exposure to ELS [177]. Bahari-Lavan et al. (2017) confirmed that ELS increases schizophrenia-like phenotypes in mice and humans. They demonstrated that the overexpression of histone–deacetylase (HDAC)1 in PFC mice neurons, but not in the hippocampus, mimics schizophrenia-like phenotypes induced by ELS. Administration of an HDAC inhibitor ameliorated these phenotypes, suggesting that HDAC1 inhibition could be a therapeutic approach for schizophrenia. In addition, mice subjected to ELS exhibited an increased HDAC1 level in blood samples, and this effect was also observed in blood samples from patients with schizophrenia who had experienced ELS. Therefore, HDAC1 inhibition should be considered a therapeutic approach for treating schizophrenia. Measuring HDAC1 levels in blood samples may allow patient stratification and individualized therapy [177].

These findings underscore the ELS’s critical impact on long-term mental and physical health. ELS triggers a cascade of physiological changes, including epigenetic modifications, that predispose individuals to heightened stress sensitivity and various psychiatric disorders. Understanding these mechanisms is crucial for developing targeted interventions to mitigate the adverse effects of ELS and improve mental health outcomes.

## 6. Neurodegenerative Changes and Early-Life Stress, Aging, and Environmental Toxins

Neurodegenerative changes stem from factors such as ELS, aging, and environmental toxins, each uniquely impacting the nervous system and potentially exacerbating one another. ELS, in particular, can induce lasting gene expression changes through epigenetic mechanisms like DNA methylation, histone modification, and non-coding RNA, shaping cell function and influencing vulnerability to environmental factors [23]. These epigenetic changes during early development can lead to structural alterations in the hippocampus, PFC, and amygdala, resulting in cognitive deficits, impaired emotional regulation, and increased susceptibility to psychiatric disorders such as depression and anxiety, as well as neurodegenerative diseases like Alzheimer’s and Parkinson’s, later in life.

Different studies indicate a notable, yet variable, increase in the risk of neurodegenerative diseases among individuals with ELS. For instance, some studies suggest that ELS can increase the overall risk of neurodegenerative disorders by 1.5 to 3 times, depending on the disease and the specific type and timing of stress experienced [178,179,180]. One longitudinal study found that individuals with significant ELS had an OR of 1.6 (95% CI: 1.2–2.2) for developing dementia [181]. Additionally, research suggests that ELS may exacerbate the risk of neuroinflammation, which is a known risk factor for neurodegenerative disorders like AD. The OR for heightened neuroinflammation in individuals with ELS is reported to be 2.0 (95% CI: 1.5–2.6) [182].

Although neurodegenerative changes due to ELS, aging, and environmental toxins share common pathways like oxidative stress, neuroinflammation, and impaired cellular function, each has unique triggers and mechanisms. ELS primarily affects gene expression and neurodevelopment; a natural decline in physiological processes marks aging, and environmental toxins directly inflict neural damage. Understanding these differences is crucial for developing targeted interventions for neurodegenerative diseases.

### Early-Life Stress in Neurodegenerative Disorders

Parkinson’s disease is characterized by impaired movement control due to the loss of DA-producing neurons, particularly in the substantia nigra pars compacta (SNpc). It is also associated with non-motor symptoms, such as depression, anxiety, and dementia [183]. A recent study suggested a bidirectional relationship between depression and PD, indicating that individuals with depression may have a higher risk of developing PD [184]. Maternal separation accelerated the PD progression in a 6-OHDA-induced rat model in addition to inducing a depression-like behavior characterized by a decreased sucrose preference [185].

Interestingly, MS can induce a PD phenotype in rats with aging associated with alterations of the nigrostriatal dopaminergic system without any neurotoxin intervention [186]. Indeed, neuroimaging and histochemical analysis revealed a reduced expression of DA-related markers, DAT, and TH in the striatum of maternal-separated rats at 36 and 60 weeks. In the same rats, transcriptome analysis revealed distinct gene expression patterns, particularly in pathways associated with dopaminergic synapses and processes related to locomotion. These findings suggest that depression induced by ELS in rats mirrors some prodromal features of PD during natural aging.

Conversely, while it is well-documented that stressors accelerate disease progression in animal models of PD, there is limited understanding of how individuals with PD respond to stressful life events [187]. In stressful situations, PD motor symptoms worsen, and dopaminergic medication is less effective; this may be because striatal DA, which helps respond to stress, is insufficient in PD patients [188]. Resilience, the ability to successfully adapt to stressors and recover mental health during adversity, involves personal or contextual factors known as resilience factors, which are crucial for preventing stress-related issues. Both stress-reactivity and resilience can be evaluated under stressful circumstances. Therefore, van der Heide et al. (2024) used the COVID-19 pandemic as a natural environment to study these two parameters. The authors described stressor-reactivity (SR) as how much a person’s mental health is affected by COVID-19 stressors at a particular time. More resilient individuals showed low SR scores over six months. The study examined resilience and risk factors for stressor-reactivity in PD patients during the COVID-19 pandemic. It assessed whether SR was linked to different rates of symptom progression. They discovered that pre-existing anxiety, severe non-motor symptoms, and rumination predicted high SR (risk factors) [188].

On the other hand, factors like quality of life, cognitive abilities, and social support predict low SR (resilience factors). In PD individuals, high SR did not lead to a faster progression of motor symptoms during the pandemic. Still, it was associated with an increase in depressive symptoms [188]. These findings suggest that non-motor symptoms, such as cognitive and psychiatric issues, were more predictive of resilience in PD patients than motor symptoms. As a result, motor symptoms did not worsen due to stressor-reactivity during the pandemic, but depressive symptoms increased explicitly in those who were highly stress-reactive.

HPA axis deregulations have also been observed in AD patients as a consequence of amyloid toxicity (see Brureau study, 2013 [189]). AD, an age-related and progressive neurodegenerative disorder, is characterized by amyloid β (Aβ) plaques and tau tangles, leading to memory loss and psychological issues such as anxiety [190]. Brureau et al. (2013) demonstrated that intracerebroventricular injection of amyloid- β_25–35_ peptide (Aβ_25–35_) in adult rats, a validated acute model of AD, induced hyperactivity in the HPA axis and increased levels of GRs and MRs in brain areas linked to HPA axis function. This resulted in behavioral changes typical of limbic system dysfunction [189]. They observed GR function, location, and number alterations, characterized by an imbalanced MR/GRs ratio and disrupted nucleocytoplasmic shuttling. These results suggest that the Aβ_25–35_ toxicity affects the axis’s response to stress [189].

As described above for PD, ELS is also used to advance AD research. Recent studies have shown that neonatal MD in mice disrupts glymphatic development, a brain-wide macroscopic system for the efficient clearance of harmful metabolites such as Aβ, impairs α-synuclein clearance from the brain and aggravates susceptibility to AD later in life [191].

In conclusion, there is a very complex link between ELS and neurodegenerative diseases like PD and AD. The impact of stress on the progression and severity of these diseases is evident through various pathways, including alterations in the dopaminergic system and HPA dysregulation. Understanding these mechanisms not only highlights the importance of managing stress to mitigate the risk and progression of neurodegenerative diseases but could develop targeted therapeutic interventions that address these specific stress-related pathways.

## 7. Understanding the Impact of Early-Life Stress on Glioblastoma Progression

The previous paragraphs illustrated ELS’s significant and long-lasting effects on various physical and mental health aspects. While studies on the direct link between ELS and glioblastoma are limited, there is growing interest in understanding how ELS might influence the development and progression of glioblastoma, a highly aggressive form of brain cancer. Glioblastoma (GBM), also known as GBM multiform, is the most common malignant primary brain tumor in adults. Despite aggressive treatment approaches, including surgery, radiation therapy, and chemotherapy, the prognosis for patients with glioblastoma remains dismal, with a median survival of approximately 12 to 18 months from the time of diagnosis [192]. Physical and psychological stress is common in patients with GBM due to diagnosis, repeated treatments, and tumor recurrence, leading to a higher rate of depression compared to both the general population and other cancer patients [193].

As previously discussed, ELS can disrupt the HPA axis, leading to the prolonged release of cortisol and other stress hormones. Elevated cortisol levels can weaken the immune system’s ability to control inflammation, potentially causing long-lasting neuroinflammation. ELS also triggers chronic microglia activation, which releases pro-inflammatory cytokines, sustaining an inflammatory state in the brain. Additionally, ELS compromises the integrity of the blood–brain barrier, allowing peripheral immune cells and inflammatory molecules to enter the brain and worsen neuroinflammation. Astrocytes can also regulate CNS inflammation and neurodegeneration through various mechanisms, such as neurotoxicity, modulation of microglial activities, recruitment of inflammatory cells into the CNS, and their metabolic cascade.

While the connection between ELS and GBM is not fully understood, it is plausible that ELS-induced chronic neuroinflammation could play a crucial role in promoting tumor growth by providing essential growth factors and inflammatory cytokines. Inflammatory cells within the tumor microenvironment enhance glioblastoma cell proliferation, invasion, and drug resistance [194]. At the same time, Perelroizen et (2022) identified a mechanism by which astrocytes also play a crucial role in controlling the pathogenicity of glioblastoma by reprogramming the immunological properties of the tumor microenvironment and sustaining the metabolic landscape necessary for tumor survival. They demonstrated that astrocyte-derived cholesterol is crucial for glioma cell survival, emphasizing the significance of targeting astrocytic cholesterol efflux, mainly via sterol transporters ABCA1, in halting tumor progression [195]. Microenvironmental stress within the extracellular matrix (ECM) may also impact tumor malignancy. In 2022, Khoonkari et al. demonstrated that increased production of various ECM components can cause stiffening in the brain. This mechanical stress modifies mechanosensing signaling pathways, including the Hippo/YAP pathway, CD44 pathway, and actin skeleton signaling, which may influence glioblastoma cell proliferation, migration, and invasion [196].

Long-lasting epigenetic changes triggered by ELS may also influence the interplay between ELS and glioblastoma. ELS-altering DNA methylation and histone modifications can affect gene expression patterns related to cell growth, apoptosis, and DNA repair, potentially contributing to the development and progression of glioblastoma. Janusek et al. (2017), analyzing African American men, reported that exposure to trauma and adversity during early life amplified the adult pro-inflammatory response to stress by the hypomethylation of IL-6 promoter [197]. Correlated to this, an increase in the expression level of hypomethylated genes in GBM has been observed compared with that in normal brain tissue, including that of IL-6 [197].

Additionally, Pfau et al. (2019) demonstrated that the miR-106b~25 cluster mediates inflammatory and behavioral responses to repeated social defeat stress mouse models [198]. Accordingly, the miR-106b~25 cluster modulates cancer stem cell characteristics and might promote resistance to anticancer therapies. An increased expression of miR-25 inhibited the proliferation of glioblastoma cells with functional p53 protein. Moreover, p53 negatively regulated miR-106b~25 cluster expression by inhibiting its transcriptional inducers E2F1 and MYC. Suh et al. (2012) described a complex autoregulatory circuit involving p53, E2F1, MYC, and miR-106b~25 cluster [199].

A meta-analysis study inversely correlated SES with chronic inflammation, indicating that individuals with lower SES showed higher levels of systemic inflammatory markers, like C-reactive protein (CRP) and IL-6. Instead, using a multi-level modeling approach, Needham et al. (2015) also correlated socioeconomic status with the DNA methylation status of stress and inflammation-related genes, such as NLRP12 [200]. By next-generation sequencing database, NLRP12 expression increased in glioma cells, and its knockdown attenuated GBM cell invasion and inhibited cell migration [201].

Finally, ELS may influence glioblastoma progression through stress hormones like GCs, noradrenaline, and DA and their receptor systems. These hormones can interact with tumor cells and the tumor microenvironment, promoting tumor growth and resistance to treatment.

Chronic stress, but we cannot exclude a similar mechanism in ELS, has recently been discovered to activate the PI3K/Akt signaling pathway by binding GCs and noradrenaline to glucocorticoid and β-adrenergic receptors. This activation leads to glioma cell proliferation in vitro and tumor growth in vivo, accompanied by elevated serum levels of GCs and noradrenaline [202,203,204,205]. The noradrenaline upregulates CD147 expression via the β-adrenergic receptor (βAR)-β-arrestin1-ERK1/2-Sp1 pathway, and this increase enhances MMP-2 secretion and extracellular lactic acid levels, promoting glioma cell invasion and metastasis under psychological stress [206].

It is worth noting that antipsychotic, antidepressant, and mood-stabilizing drugs, when used in GBM patients, have demonstrated properties that are anti-tumor, anti-metastatic, anti-angiogenic, and anti-proliferative [192]. These medications, including risperidone, haloperidol, chlorpromazine, trifluoperazine, and pimozide, hinder the malignant advancement of glioma by primarily targeting the DAD2R signaling pathways. These pathways are involved in cell proliferation, apoptosis, and invasion/migration [207,208] or in enhancing the sensitivity of glioma cells to conventional chemotherapeutic drugs or radiotherapy [209,210]. In 2023, Wang and collaborators discovered that long-term stress boosts glioblastoma by increasing DA and DRD2 levels through the DRD2/ERK/β-catenin pathway and the DA/ERK/TH regulatory loop [211]. Additionally, they demonstrated that combining pimozide, a specific DRD2 inhibitor, and temozolomide exerts a more potent anti-tumor effect in GBM [211]. Therefore, the possibility of acting on different pathways in combination with conventional treatments such as chemotherapy or immunotherapy emerges as a rational strategy to increase therapeutic effectiveness against glioma. Additionally, the ongoing discovery of novel targets holds the potential for advancing the formulation of strategies aimed at shielding glioma patients from the adverse effects of stress on cancer progression [192].

ELS’s impact on the development and progression of glioblastoma must be considered. By understanding how ELS affects neuroinflammation, epigenetic modifications, and hormone release, we can identify individuals at higher risk and develop targeted monitoring and early intervention strategies. This knowledge can lead to developing preventive measures, such as stress reduction programs, especially for at-risk populations. Personalized treatments based on an individual’s stress history could significantly enhance the effectiveness of therapeutic interventions. Moreover, integrating psychological and social support into cancer care can address the psychological factors influencing tumor progression and improve patient outcomes. By studying the relationship between ELS and glioblastoma, we encourage interdisciplinary research and pave the way for innovative treatments and discoveries that could apply to other cancers and stress-related health issues.

## 8. Early-Life Stress: Prevention, Intervention, and Therapeutic Strategies

As this review extensively describes, ELS can profoundly affect an individual’s physical, emotional, and psychological well-being. Dealing with ELS demands a well-rounded approach encompassing prevention, intervention, and therapeutic strategies.

### 8.1. Prevention and Intervention Strategies

It is vital to prioritize parental education and support to effectively leverage research on adverse childhood experiences (ACEs) to prevent them in children. As extensively revised by Narayan et al. (2021) [212], this can be accomplished through a multistep approach. Providing accessible programs that offer continuous education on child development, stress management, and effective parenting techniques is crucial. Trained professionals visiting homes to offer personalized support and education to parents, especially those at high risk of stress, are also essential. In addition, it is important to include mental health screenings and support for parents in pediatric and maternal health services to detect and address parental stress early [213]. For example, the Nurse-Family Partnership (NFP) is a program where nurses visit first-time, low-income mothers during pregnancy and the first two years of the child’s life. Long-term studies indicate that NFP improves maternal and child health outcomes, reduces child abuse and neglect, and enhances children’s cognitive and emotional development. Nurses guide prenatal care, parenting skills, and family planning to create a supportive and educational environment for young mothers [214].

Another critical intervention is the establishment of accessible community centers that offer various services, such as parenting classes, support groups, recreational activities, and emergency assistance. Equally important is creating and promoting volunteer programs and peer support networks where parents can share experiences, advice, and support. Introducing educational programs in schools, workplaces, and community organizations to increase awareness and understanding of ELS and its prevention is equally crucial. Furthermore, early identification and support are imperative. Incorporating ELS assessments into routine pediatric visits ensures early identification and intervention for at-risk children. Developing school-based programs that focus on mental health and well-being and providing early support and resources to students is essential. The program includes school-based counseling, family therapy sessions, and teacher training on trauma-informed practices. Implementing regular screenings in childcare centers and preschools to identify and address early signs of stress and trauma is also critical [213]. For instance, the Chicago Child–Parent Center (CPC) program is an early childhood intervention program that provides comprehensive educational and family support services to children from low-income families. Longitudinal studies have shown that CPC participants have higher educational attainment, lower rates of juvenile arrest, and better social-emotional outcomes. The program includes high-quality preschool education, family support services, and parental involvement in the educational process [215,216].

Enhancing prevention and intervention strategies can provide more effective and accessible support for children and families experiencing ELS, ultimately promoting healthier and more resilient communities.

### 8.2. Psychological Interventions

Therapeutic interventions are essential to address the impact of ELS, lessen its effects, and enhance resilience and recovery. One is psychological interventions such as cognitive behavioral therapy (CBT). CBT helps individuals recognize and alter negative thought patterns and behaviors that stem from ELS. A meta-analysis study has been shown to reduce symptoms of depression and anxiety, expected outcomes of ELS in victims of sexual abuse and war trauma, and patients aged more than seven years [217]. Another approach includes trauma-focused therapy, such as cognitive processing therapy (CPT), prolonged exposure Therapy (PE), eye movement, desensitization, and restructuring (EMDR) [218], which is effective in reducing PTSD symptoms and improving emotional regulation.

A study conducted in the central region of Israel observed pregnant women at community and hospital medical centers, highlighting the importance of skin-to-skin contact between mothers and infants after birth to reduce posttraumatic stress symptoms following childbirth. This effect is particularly significant for women who have undergone a cesarean section [219]. Furthermore, skin-to-skin contact has been shown to alter biomarker levels, leading to lower cortisol levels in both infants and parents and increased oxytocin levels. Oxytocin, commonly known as the “love hormone”, promotes bonding and helps reduce stress and anxiety [220,221]. Moreover, direct skin contact can influence DNA methylation patterns, which control gene expression. The NR3C1 gene, responsible for encoding the glucocorticoid receptor, has been extensively researched and has consistently shown epigenetic modifications and changes in expression when subjected to ELS, leading to an increased vulnerability to stress-related illnesses in mice [222]. Human studies have supported these findings, indicating that early skin-to-skin contact can result in enduring alterations in stress-related gene expression and overall health outcomes. The relative mRNA expression of the NR3C1 gene was significantly decreased in infants with skin-to-skin contact.

ELS can influence the expression of specific genes, such as corticotropin-releasing hormone receptor 2 (CRH R2) and the serotonin transporter gene (SLC6A4). This can lead to long-term neurobehavioral impairments in both rodents and humans [223,224]. The expression of CRH R2 is correlated with HPA axis reactivity and parameters of mother-child interaction at six months of corrected age.

In summary, skin-to-skin contact is a simple but highly impactful practice. It immediately reduces stress for infants and parents and brings positive epigenetic changes. These modifications can improve stress resilience and overall health, highlighting the significance of early physical bonding experiences.

### 8.3. Pharmacological Interventions

Therapeutic strategies for neuropsychiatric symptoms from ELS, such as depression and anxiety, include psychological interventions and pharmaceutical management. Studies on animals show that 30-day fluoxetine (Prozac) treatment reversed ELS-induced depression-like behaviors, particularly in terms of sucrose preference. Fluoxetine also restores serotonin transporter (SERT) levels in the amygdala and norepinephrine transporter (NET) levels in the mPFC and hippocampus. Compared to vortioxetine, which reversed the decreased expression of VMAT2 in the NAc, fluoxetine selectively targets SERT. Additionally, fluoxetine, but not vortioxetine, restored NET levels in the hippocampus. These findings highlight the role of monoamine transporters in ELS-induced depression and provide insights into depression treatment in women. Nutrition also plays a crucial role in addressing ELS. Recent studies suggest that adding fish oil supplements to antidepressants like fluoxetine can reduce ELS-related depressive and anxiety symptoms. Research on maternal-separated rats shows fish oil lowers corticosterone levels, improves serotonin turnover, and alters the gut–brain axis, demonstrating the potential of nutraceuticals and pharmaceuticals to alleviate ELS effects [225]. 

## 9. Discussion

The profound impact of ELS on mental and physical health underscores the critical urgency for early interventions and preventive measures. ELS induces substantial changes in brain structure and function, exerting long-lasting effects on an individual’s psychological well-being. In this review, we emphasize the significance of utilizing animal models such as maternal separation, maternal deprivation, and limited bedding and nesting to gain insights into the impact of ELS. Many more animal models of ELS are not mentioned in the review, using either singular or multiple types of stressors (multi-hit), including perinatal infection, lipopolysaccharide-induced inflammation, toxin exposure, and malnutrition [226]. Such stressors can dramatically impact brain development and increase the risk of neuropsychiatric disorders. While acknowledging the limitations of animal models, researchers must carefully translate these findings to human contexts to develop targeted interventions that alleviate the enduring effects of early adversity in humans.

Furthermore, our discussions have delved into ELS’s far-reaching and enduring impact on fundamental molecular pathways crucial for brain development, stress regulation, and mental well-being. Extensive research in this domain has unveiled the intricate neurobiological mechanisms that drive the profound changes ELS brings. Dysregulation of the HPA axis, alterations in neurotransmitter systems, and various epigenetic modifications, including DNA methylation, histone modifications, RNA modifications, and non-coding RNA activity, all play pivotal roles. Specifically, we have examined the significant impact of ELS on dopaminergic neurocircuitry, emphasizing its implications for mental health vulnerability. The influence of ELS on dopaminergic neurocircuitry is profound and multidimensional, impacting both the structural and functional aspects of the brain’s reward pathways. Moreover, the text underscores the intricate interplay between ELS, socioeconomic factors, and subsequent heath.

Understanding these mechanisms deeply opens new avenues for targeted therapies to mitigate the adverse effects of ELS. Moreover, it emphasizes the importance of creating supportive environments and implementing therapeutic strategies to foster resilience and adaptive coping mechanisms in affected individuals.

**Figure 3:**
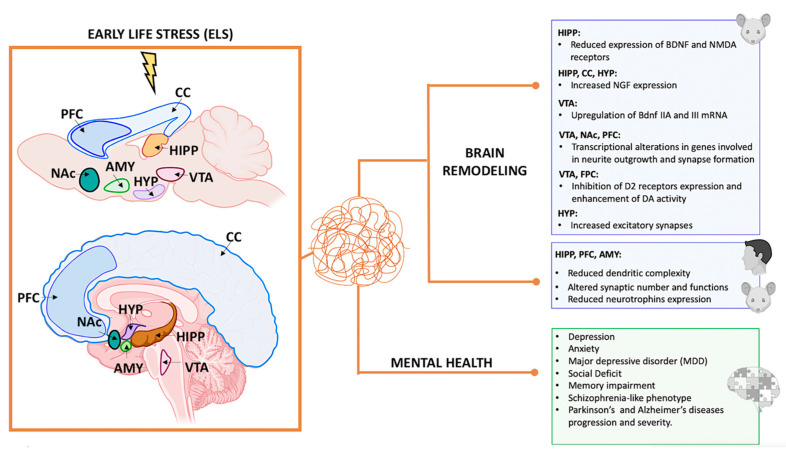
Summary of the effects of (ELS) on brain remodeling and mental health disorders in mice and humans. This figure summarizes the impact of ELS (red wavy lines) on brain remodeling and the development of mental health disorders, drawing parallels between findings in mice (top brain) and humans (bottom brain). It highlights how ELS can lead to significant changes in brain structure and function, which are associated with an increased risk of various mental health disorders. AMY: amygdala; CC: cortical cortex; FPC: frontoparietal cortex; HIPP: hippocampus; HYP: hypothalamus; NAc: nucleus accumbens; PFC: prefrontal cortex; VTA: ventral tegmental area. Illustrations in the panel were created with Biorender.com.

Research into neuronal remodeling reveals how stress during critical developmental periods can alter neuronal connectivity, synaptic plasticity, and neurogenesis. These alterations are closely linked to an increased vulnerability to mental health disorders such as anxiety, depression, and PTSD, as well as a predisposition to neurodegenerative diseases like Parkinson’s and Alzheimer’s disease in individuals with a history of ELS. Additionally, stress impacts physical health, contributing to metabolic disorders, cardiovascular disease, and even cancer (Figure 3).

Future studies should prioritize longitudinal research to elucidate further the long-term trajectories of ELS-induced neuronal remodeling and its behavioral consequences.

Understanding the complex interplay between ELS, neuroinflammation, epigenetic changes, stress hormones, and socioeconomic factors is crucial for unraveling the mechanisms through which early life experiences may influence glioblastoma progression. Targeting these mechanisms could lead to innovative therapeutic strategies to mitigate stress’s impact on cancer outcomes, thereby improving patient prognosis and quality of life. This section highlights ongoing research efforts to elucidate the connections between ELS and glioblastoma progression, underscoring the importance of considering psychosocial factors in cancer research and treatment strategies.

Finally, we have discussed how a combination of psychological and pharmacological interventions can provide a comprehensive approach to treating the complex effects of ELS. By addressing both the psychological and biological aspects of trauma, these strategies aim to enhance resilience, alleviate symptoms, and promote long-term recovery in individuals affected by ELS. Going beyond traditional psychotherapeutic approaches, the article examines the therapeutic benefits of physical bonding experiences, explicitly focusing on skin-to-skin contact between mothers and infants. This practice (a) Reduces post-traumatic stress symptoms in mothers, particularly after cesarean sections; (b) Alters biomarker levels, including lowering cortisol (stress hormone) levels in both infants and parents and increasing oxytocin (bonding hormone) levels; (c) Influences DNA methylation patterns, potentially leading to long-term changes in stress-related gene expression and overall health outcomes.

This review also examines pharmacological interventions to address the biological aspects of disorders related to ELS. These interventions target different neurotransmitter systems and biological pathways implicated in stress response and emotional regulation, providing personalized treatment options. Additionally, novel approaches such as psychedelic-assisted psychotherapy, involving the controlled use of substances like MDMA (ecstasy) and psilocybin (magic mushrooms), are explored to facilitate therapeutic breakthroughs in patients with PTSD. These substances are believed to enhance emotional processing and increase neuroplasticity in the brain. The article also highlights the potential of allopregnanolone, a neurosteroid that acts as a potent GABAergic neuromodulator and has shown promise in preclinical studies for modulating stress responses and inflammation. It emphasizes the importance of continued research into innovative therapies and personalized treatment approaches for improving outcomes and quality of life for those impacted by early-life adversity.

## 10. Conclusions

In conclusion, deepening our understanding of the extensive impacts of ELS and devising tailored interventions is paramount. This effort is crucial for improving the well-being and quality of life of individuals affected by ELS. Continuous research in these domains is essential for developing effective strategies to mitigate early adversity’s enduring effects and foster overall health and wellness across communities.

## Data Availability

Not applicable.
